# Pandemic-Triggered Adoption of Telehealth in Underserved Communities: Descriptive Study of Pre- and Postshutdown Trends

**DOI:** 10.2196/38602

**Published:** 2022-07-15

**Authors:** Pei Xu, Matthew Hudnall, Sidi Zhao, Uzma Raja, Jason Parton, Dwight Lewis

**Affiliations:** 1 Department of Systems and Technology Auburn University Auburn, AL United States; 2 Department of Information Systems, Statistics, and Management Science University of Alabama Tuscaloosa, AL United States

**Keywords:** telehealth services, telemedicine, COVID-19, rural communities, mental health, Medicaid, health service, telehealth, health care service, health care facility, undeserved community, undeserved population, health claim, technology adoption, health insurance

## Abstract

**Background:**

The adoption of telehealth services has been a challenge in rural communities. The reasons for the slow adoption of such technology-driven services have been attributed to social norms, health care policies, and a lack of infrastructure to support the delivery of services. However, the COVID-19 pandemic–related shutdown of in-person health care services resulted in the usage of telehealth services as a necessity rather than a choice. The pandemic also fast-tracked some needed legislation to allow medical cost reimbursement for remote examination and health care services. As services return to normalcy, it is important to examine whether the usage of telehealth services during the period of a shutdown has changed any of the trends in the acceptance of telehealth as a reliable alternative to traditional in-person health care services.

**Objective:**

Our aim was to explore whether the temporary shift to telehealth services has changed the attitudes toward the usage of technology-enabled health services in rural communities.

**Methods:**

We examined the Medicaid reimbursement data for the state of Alabama from March 2019 through June 2021. Selecting the telehealth service codes, we explored the adoption rates in 3 phases of the COVID-19 shutdown: prepandemic, pandemic before the rollout of mass vaccination, and pandemic after the rollout of mass vaccination.

**Results:**

The trend in telemedicine claims had an opposite pattern to that in nontelemedicine claims across the 3 periods. The distribution of various characteristics of patients who used telemedicine (age group, gender, race, level of rurality, and service provider type) was different across the 3 periods. Claims related to behavior and mental health had the highest rates of telemedicine usage after the onset of the pandemic. The rate of telemedicine usage remained at a high level after the rollout of mass vaccination.

**Conclusions:**

The current trends indicate that adoption of telehealth services is likely to increase postpandemic and that the consumers (patients), service providers, health care establishments, insurance companies, and state and local policies have changed their attitudes toward telehealth. An increase in the use of telehealth could help local and federal governments address the shortage of health care facilities and service providers in underserved communities, and patients can get the much-needed care in a timely and effective manner.

## Introduction

The COVID-19 pandemic has stress-tested worldwide health systems like no other event in modern times. Requirements for limited travel, social distancing, and business closures have adversely impacted many segments of society in order to help mitigate the spread of the virus. In this context, telemedicine has been promoted and expanded to reduce the risk of viral transmission [1].

Telemedicine enhances the delivery and availability of health care services. As a result, during the COVID-19 pandemic, telemedicine should be a vital tool in providing care, while keeping patients and health professionals safe [2]. Telemedicine, as an effective tool to increase patients’ accessibility to health services, may effectively reduce medical costs and improve patients’ quality of life [3]. The future benefits of telemedicine include its cost-effectiveness, its ability to expand specialty services, and its potential to help alleviate looming physician shortages [4].

Prior to COVID-19, telehealth in Alabama, USA, was broadly available but seldom utilized. Telehealth equipment was available in all 67 county health offices, most hospitals, and many physicians throughout the state. Within the Medicaid population, prepandemic telehealth utilization averaged around 1000 people, with 1.3 sessions per person within any given month [5]. This was among a Medicaid population that consists of approximately 1.2 million persons on an annual basis. This low utilization rate was due to a myriad of factors related to billing practice, patient/physician comfort, and familiarity with using the technology [6,7].

On April 3, 2020, Alabama Governor Kay Ivey issued a stay-at-home order to help curb the increasing spread of the virus [8]. This order, in conjunction with the widespread fear of contracting the virus during the prior months, created a unique environment for the necessitated rapid adoption of telemedicine. At the same time, insurance companies adjusted their policies so that telemedicine visits became reimbursable at approximately the same rates as in-person visits [9]. The changes to reimbursement rules coupled with the necessitated modality created the environment through which telemedicine was given an opportunity to shine.

Prior studies have highlighted the importance of studying the granularity of telemedicine adoption using subpopulation analyses. For instance, Chu et al [10] suggested that further studies are required “to assess the potential barriers to telemedicine experienced by rural populations compared to those experienced by urban populations and the impact of telemedicine compared to that of in-person care on other forms of health care utilization, outcomes, and quality of care among vulnerable and at-risk patient groups in the rural population.” Monaghesh and Hajizadeh [2] proposed that researchers can “examine the effectiveness of using telehealth approaches in different health areas, especially in the field of home nursing the elderly who are high-risk people in the community. It is also highly recommended to use this technology in the field of psychiatry as it does not require in-person visits.” As a response to these calls, this paper aims to examine the prior utilization rates of telemedicine among the Alabama Medicaid population and compares those rates stratified by demographics to the rates during the COVID-19 pandemic.

## Methods

### Data

Since 2014, the Institute of Data and Analytics (IDA) at the University of Alabama has had a consulting contract with Alabama Medicaid, in which the IDA supports Alabama Medicaid’s analytics group. To fulfill this contract, the IDA has maintained a secure copy of all Alabama Medicaid enrollment and claims data since 2010. These data include specific billing/claim information for all enrolled in Alabama’s Medicaid program. Annually, approximately 1 (25%) of every 4 Alabamians has some form of Medicaid coverage during the year. Medicaid insurance services are made available to low-income populations, as well as those who are disabled, at no cost to participants.

Data from the procedure and provider tables within the Medicaid database were extracted that contained corresponding billing codes indicating actions had taken place over telemedicine. Claims information within the Medicaid database can lag behind for up to 6 months since facilities and practitioners may not always bill in a timely manner. However, over 80% of claims are received within a month from the time of service. The variables extracted included the year and month of the service, the county where the service took place (provider location), the gender of the patient, the age range of the patient corresponding to US Census age groups, provider/procedure information, modality of service (in person vs telehealth), and total billing costs paid. Procedures were further grouped by type of procedure and group of procedure, and providers were further grouped by type of provider as well as the specialty of the provider. These variables were aggregated, and calculations of counts of unique persons, counts of claims, and total cost of claims for each level of aggregation were calculated. The data represent a complete sampling of the Alabama Medicaid records from March 1, 2019, through June 30, 2021. The data were extracted from the August 2021 data backup of the Alabama Medicaid data, which included transactions through July 2021 and represented approximately 80% of all claims through June of that year. In total, 94,254,599 patient claims covered by Alabama Medicaid at some point during the analysis period are represented in the telehealth utilization data, along with a total of about US $5 billion across 738 procedure types from 27 provider categories across 97 specialties.

The COVID-19 tracking reports were downloaded from the COVID Tracking Project [11], which collects and publishes the most comprehensive data about COVID-19 in the United States. We also collected the COVID-19 vaccination progress report for Alabama from the USAFacts website [12]. Lastly, a classification of areas as being rural or urban at the county level was obtained from the Alabama Rural Health Association [13].

### Analysis

**Figure 1:**
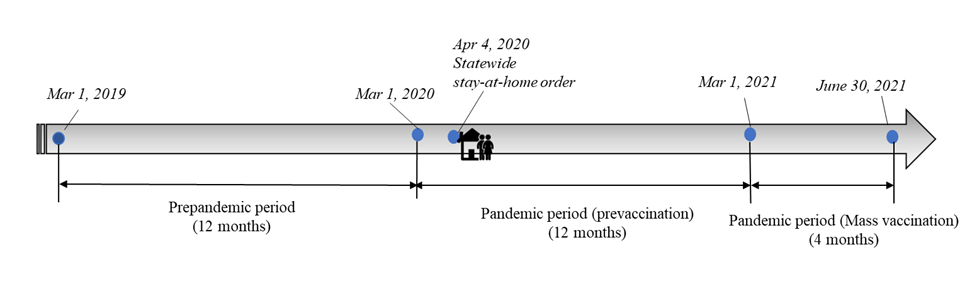
Illustration of study periods.

The cumulative time frame of analysis was from March 1, 2019, through June 30, 2021, as shown in Figure 1. Alabama announced the first known cases of coronavirus in March 2020; therefore, the time frame being considered as pre-COVID-19 was the period from March 1, 2019, to February 29, 2020. Broad COVID-19 vaccine availability in Alabama occurred during March 2021, so the time frame from March 1, 2021, to June 30, 2021 was analyzed separately as a mass vaccination condition. The core pandemic time frame was characterized by March 1, 2020, through February 28, 2021. This allowed for 12 months of data prior to COVID-19, 12 months of data during the pandemic, and 4 months of analysis of the time frame when vaccines were widely available.

We compared the characteristics of patients who utilized and did not utilize the telemedicine service in each period. For each telemedicine usage modality, chi-square tests were conducted to assess the distribution of services and patient characteristics (eg, age group, gender, race, level of rurality, and service provider type) across different study periods. We then calculated the rate of telemedicine usage for the various characteristics for each month in the study periods.

### Ethical Considerations

Institutional board approval was sought and obtained for this project from the University of Alabama's ethics board (UA IRB protocol #21-05-4661). The full study protocol is available through the institution or by contacting the authors. Additionally, the research was vetted and approved by Alabama Medicaid’s internal research review process.

## Results

### Overview

The data set contained 94,254,599 Medicaid claims from March 1, 2019, through June 30, 2021, in the state of Alabama. For each period, we reported the rate of telemedicine usage, which increased from 0.12% (n=55,613) in the prepandemic period to 3.43% (n=1,141,282) and 1.85% (n=254,807) in the subsequent 2 periods, respectively, as seen in Table 1. In addition, the second period (ie, the first year after the onset of the pandemic) saw the lowest average monthly Medicaid claims (2,774,171 claims per month) but the highest average monthly telemedicine Medicaid claims (95,107 claims per month) among the 3 study periods. Such an opposite trend was partly due to the statewide stay-at-home orders in that period.

**Figure 2:**
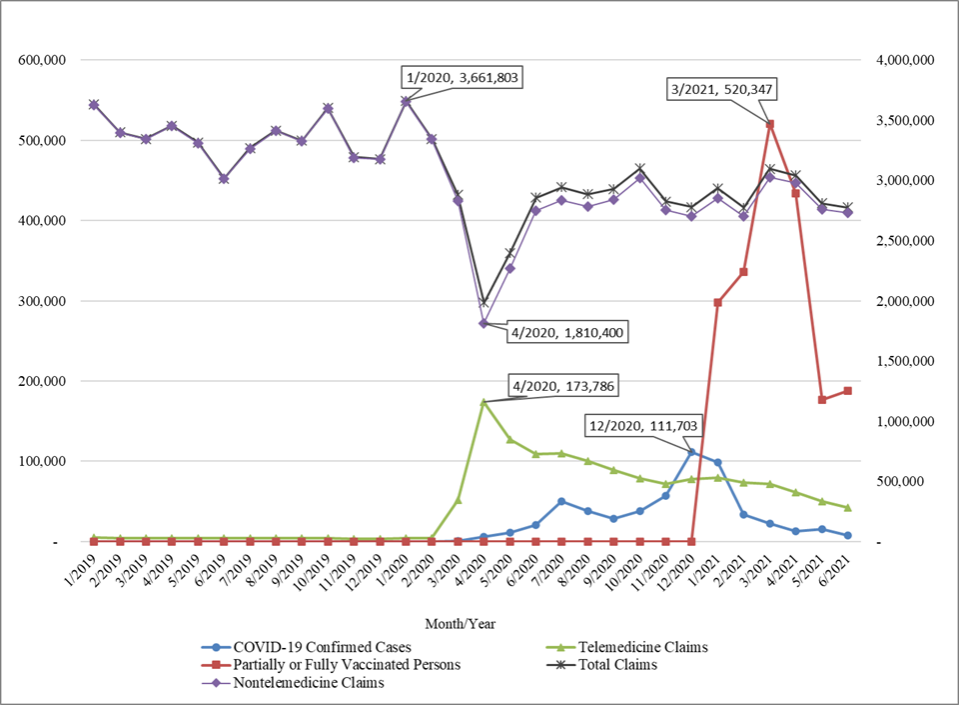
Overview of monthly COVID-19 cases, vaccination, and telemedicine usage in Alabama. The “Total Claims” and “Nontelemedicine Claims” series are plotted on the right-hand axis, while the other data series are plotted on the left-hand axis.

Figure 2 provides an overview of monthly changes in new COVID-19 confirmed cases, vaccination progress, and telemedicine usage in Alabama. As can be seen in the figure, the nontelemedicine claims and total claims dramatically dropped since March 2020. Both lines reached the valley when the state issued a stay-at-home order in April 2020, while at the same time, telemedicine usage reached its peak point. The monthly claims of telemedicine usage gradually decreased during the months after April 2020, but the number of monthly claims was still at a relatively high level at the end of our study period (ie, June 2021). This is an indication that telemedicine usage is still popular, even after the rollout of mass vaccination and the relaxation of the COVID-19–related executive orders. Interestingly, the monthly new confirmed cases peaked in December 2020, but neither telemedicine nor nontelemedicine claims exhibited any obvious changes during that period. This is possibly due to people having become accustomed to the new norm brought by the pandemic.

In Table 2 (Multimedia Appendix 1) and Table 3 (Multimedia Appendix 2), we summarize the characteristics of Medicaid claims in the 3 periods (ie, before the pandemic, after the onset of the pandemic, and after the rollout of mass vaccination). Monthly Medicaid claims are reported along with the proportions for the groups within each classification. The sparklines, with the lowest and highest values marked in blue dots, were used to visualize the trend in claim counts over the 3 periods. Chi-square tests were performed to assess the distribution of various characteristics of patients who used telemedicine (age group, gender, race, level of rurality, and service provider type) across the 3 periods. These chi-square tests all resulted in a *P* value <.001. Applying the correction for multiple comparisons, we calculated the Bonferroni correction α values for each test, and the smallest adjusted α value was 0.05/42=0.0012, suggesting that there were significant shifts in the constitution of telemedicine and nontelemedicine usage in terms of the aforementioned patient/service characteristics classifications. The trend in the total number of Medicaid claims was similar to nontelemedicine Medicaid claims, as the latter made up a large proportion of the former (see Table 1).

**Table 1:** Medicaid claims in each study period.

COVID-19 periods	Time frames	Total Medicaid claims			Average monthly Medicaid claims		
		Telemedicine, n (%)	Nontelemedicine, n (%)	Total, N	Telemedicine, n (%)	Nontelemedicine, n (%)	Total, N
Prepandemic	March 1, 2019-February 29, 2020	55,613 (0.12)	47,110,415 (99.88)	47,166,028	4634 (0.11)	3,925,868 (99.89)	3,930,502
Pandemic (prevaccination rollout)	March 1, 2020-February 28, 2021	1,141,282 (3.43)	32,148,768 (96.57)	33,290,050	95,107 (3.43)	2,679,064 (96.57)	2,774,171
Pandemic (postvaccination rollout)	March 1, 2021-June 30, 2021	254,807 (1.85)	13,543,714 (98.15)	13,798,521	63,702 (1.85)	3,385,929 (98.15)	3,449,630

**Table 2:** Monthly telemedicine and nontelemedicine Medicaid claims grouped by period and patient demographics.

Variables		Monthly nontelemedicine Medicaid claims, n (%)			Monthly telemedicine Medicaid claims, n (%)		
		Period 1	Period 2	Period 3	Period 1	Period 2	Period 3
**Age (years), *P*<.001**							
	0-17	1,621,283 (41.30)	1,073,173 (40.06)	1,500,784 (44.32)	1733 (37.39)	51,071 (53.70)	35,601 (55.89)
	18-29	462,397 (11.78)	332,697 (12.42)	444,775 (13.14)	457 (9.87)	9868 (10.38)	6693 (10.51)
	30-39	345,943 (8.81)	249,813 (9.32)	312,790 (9.24)	557 (12.01)	9929 (10.44)	6617 (10.39)
	40-49	316,603 (8.06)	219,858 (8.21)	268,471 (7.93)	578 (12.48)	7892 (8.30)	5153 (8.09)
	50-64	680,963 (17.35)	460,029 (17.17)	525,477 (15.52)	1000 (21.57)	12,617 (13.27)	7795 (12.24)
	65-74	284,218 (7.24)	197,950 (7.39)	195,061 (5.76)	236 (5.09)	2501 (2.63)	1338 (2.10)
	75-84	141,137 (3.60)	95,784 (3.58)	92,449 (2.73)	59 (1.28)	842 (0.88)	376 (0.59)
	85+	73,325 (1.87)	49,759 (1.86)	46,122 (1.36)	14 (0.31)	388 (0.41)	129 (0.20)
**Gender, *P*<.001**							
	Female	2,399,426 (61.12)	1,649,587 (61.57)	2,088,520 (61.68)	2400 (51.79)	53,068 (55.80)	35,260 (55.35)
	Male	1,517,497 (38.65)	1,020,126 (38.08)	1,282,413 (37.87)	2223 (47.98)	41,945 (44.10)	28,377 (44.55)
	Unknown	8945 (0.23)	9351 (0.35)	14,996 (0.44)	11 (0.24)	94 (0.10)	65 (0.10)
**Race/ethnicity, *P*<.001**							
	Black	1,472,153 (37.50)	1,009,310 (37.67)	1,244,880 (36.77)	1808 (39.01)	33,234 (34.94)	23,437 (36.79)
	Hispanic	114,738 (2.92)	83,240 (3.11)	121,757 (3.60)	49 (1.06)	2100 (2.21)	1317 (2.07)
	Other	671,783 (17.11)	466,643 (17.42)	608,547 (17.97)	796 (17.17)	18,772 (19.74)	12,792 (20.08)
	White	1,666,694 (42.45)	1,119,461 (41.79)	1,409,934 (41.64)	1981 (42.75)	40,991 (43.10)	26,152 (41.05)
**Rurality, *P*<.001**							
	High	1,236,296 (31.49)	828,028 (30.91)	1,025,477 (30.29)	2145 (46.28)	27,939 (29.38)	18,370 (28.84)
	Moderate	713,244 (18.17)	489,683 (18.28)	631,993 (18.67)	1025 (22.11)	17,849 (18.77)	11,990 (18.82)
	Low	1,974,916 (50.31)	1,360,294 (50.77)	1,726,747 (51.00)	1464 (31.58)	49,288 (51.82)	33,308 (51.29)

**Table 3:** Monthly telemedicine and nontelemedicine Medicaid claims grouped by period and provider type.

Provider type	Monthly nontelemedicine Medicaid claims (*P*<.001), n (%)			Monthly telemedicine Medicaid claims (*P*<.001), n (%)		
	Period 1	Period 2	Period 3	Period 1	Period 2	Period 3
American Academy of Physician Associates (AAPA)–employed physicians	23,264 (0.74)	15,387 (0.73)	21,853 (0.79)	0	355 (0.41)	300 (0.50)
Behavioral health	11,081 (0.35)	8000 (0.38)	14,764 (0.54)	18 (0.43)	4883 (5.59)	4299 (7.15)
Case manager (targeted)	33,603 (1.07)	22,280 (1.06)	23,112 (0.84)	10 (0.24)	514 (0.59)	327 (0.54)
Certified registered nurse anesthetist (CRNA)/certified registered nurse practitioner (CRNP)/nurse/midwife	271,499 (8.67)	189,631 (9.04)	262,797 (9.53)	206 (4.95)	6072 (6.95)	4374 (7.28)
Dentist	230,189 (7.35)	182,720 (8.71)	288,609 (10.47)	0	25 (0.03)	4 (0.01)
Federally qualified health clinic (FQHC)	141,790 (4.53)	93,818 (4.47)	126,486 (4.59)	83 (2.00)	4404 (5.04)	2224 (3.7)
Hospital	170,595 (5.45)	105,565 (5.03)	103,996 (3.77)	1 (0.01)	18 (0.02)	4 (0.01)
Mental health	262,456 (8.39)	155,685 (7.42)	185,949 (6.75)	3010 (72.33)	34,648 (39.64)	26,415 (43.96)
Optometrist	75,384 (2.41)	50,065 (2.39)	70,023 (2.54)	0	22 (0.02)	2 (0.00)
Physician	1,681,678 (53.73)	1,117,353 (53.29)	1,438,575 (52.18)	821 (19.73)	23,245 (26.6)	13,408 (22.31)
Podiatrist	6788 (0.22)	3152 (0.15)	3100 (0.11)	0	1 (0.001)	1 (0.001)
Psychologist	17,821 (0.57)	5551 (0.26)	8173 (0.30)	10 (0.23)	4659 (5.33)	3996 (6.65)
Rural health clinic	132,971 (4.25)	91,530 (4.37)	126,822 (4.60)	2 (0.04)	5047 (5.77)	3099 (5.16)
Therapist	70,612 (2.26)	56,159 (2.68)	82,554 (2.99)	1 (0.02)	3503 (4.01)	1645 (2.74)

#### Age

We followed the age group classification used by the Centers for Disease Control and Prevention (CDC) in reporting COVID-19 cases. We observed a significant use of telemedicine in all age groups after the onset of the pandemic. Prior to the pandemic, a greater proportion of minors (aged 0-17 years) used telemedicine (1733/4634, 37.39%) compared to other age groups, and this proportion was even more significant after the onset of the pandemic (period 2: 51,071/95,107, 53.70%; period 3: 35,601/21,234, 55.89%). For both telemedicine and nontelemedicine services, the monthly claims were the highest in the first period and lowest in the second period for all groups except for adults aged older than 65 years.

#### Gender

Prior to the pandemic, the Medicaid claims of female and male groups were almost the same (2400, 51.79%, per month vs 2223, 47.98%, per month); however, after the onset of the pandemic and mass vaccination, the number of female telemedicine visits increased at a higher rate than male visits (period 2: 53,068, 55.80%, vs 41,945, 44.10%; period 3: 35,260, 55.35%, vs 28,377, 44.55%).

#### Race/Ethnicity

We observed a significant increase in the rate of telemedicine visits among all race groups in periods 2 and 3 compared to period 1. Such growth was least substantial in the African American community. The number of African American visits changed from 1808 (39.01%) to 33,234 (34.94%) from period 1 to period 2, and the latter was about 18 times greater than the former. The number of claims of the Hispanic group changed from 49 (1.06%) to 2100 (2.21%) per month from period 1 to period 2, which is a 42-fold increase. The White community also saw a 20-fold increase in terms of monthly claims.

#### Rurality

We first categorized the counties into 3 levels of rurality according to the method developed and used by the Alabama Rural Health Association [13]. The method uses 4 variables (ie, the percentage of total employment, the dollar value of agricultural production per square mile of land, the population per square mile of land, and the population of the largest city in the county), with each variable accounting for 25 of a possible 100 points. The higher the overall score is, the more rural a county is rated (Table 4).

For patients in all types of rurality, we observed an increase in telemedicine usage after the onset of the pandemic. Interestingly, the increase was more obvious in urban patients, as the proportion of urban telemedicine–using patients changed from 31.58% (n=1464) in period 1 to 51.82% (n=49,288) in period 2 and 52.29% (n=33,308) in period 3, while the overall urban telemedicine–using patients were around 50% in all 3 periods.

**Table 4:** Alabama rural and urban counties.

Level of rurality	Counties
Highly rural	Barbour, Bibb, Blount, Bullock, Butler, Cherokee, Choctaw, Clarke, Clay, Cleburne, Coffee, Conecuh, Coosa, Covington, Crenshaw, Cullman, Dallas, DeKalb, Escambia, Fayette, Franklin, Geneva, Greene, Hale, Henry, Jackson, Lamar, Lawrence, Lowndes, Macon, Marengo, Marion, Marshall, Monroe, Perry, Pickens, Pike, Randolph, Sumter, Washington, Wilcox, Winston
Moderately rural	Autauga, Baldwin, Chambers, Chilton, Colbert, Dale, Elmore, Limestone, Russell, St. Clair, Talladega, Tallapoosa, Walker
Urban	Calhoun, Etowah, Houston, Jefferson, Lauderdale, Lee, Madison, Mobile, Montgomery, Morgan, Shelby, Tuscaloosa

#### Provider Type

**Figure 3:**
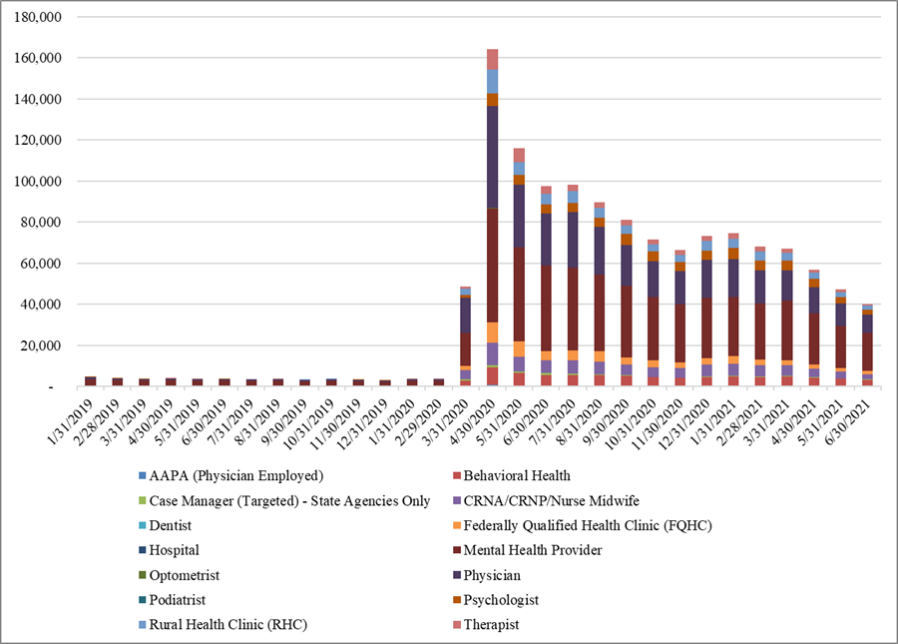
Telemedicine claims by provider type.

Table 3 (Multimedia Appendix 2) displays the monthly Medicaid claims by service type over the 3 study periods. The sparkline, with the lowest and highest values marked, indicates that the number of telemedicine claims for all service types dramatically increased in period 2 and slightly dropped in period 3. The number of a few services only slightly reduced in period 3, such as behavioral health, mental health, and psychology, suggesting a continued enthusiasm for telemedicine in these areas. In addition, mental health services accounted for about 72.33% (n=3010) of all telemedicine claims in period 1, 39.64% (n=34,648) in period 2, and 43.96% (n=26,415) in period 3. The shrinking in the mental health service proportion reveals the dramatic growth of telemedicine usage in other areas after the onset of the pandemic. Such a trend is also demonstrated in Figure 3, in which we visualize the distribution of claims across different service types.

### Rate of Telemedicine Visits

For each month within the study time frame, we reported the rate of telemedicine visits, which is defined as the count of telemedicine claims divided by the count of all claims. We then compared the rates across groups for the various characteristics.

#### Age

**Figure 4:**
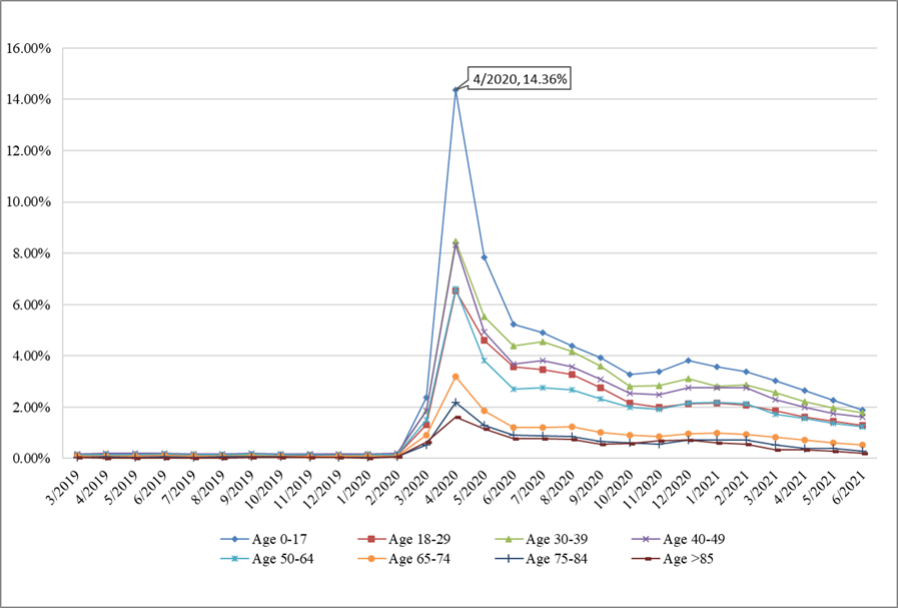
Rate of telemedicine visits by age group.

As shown in Figure 4, the rates of telemedicine visits were low (close to 0) for all age groups before the onset of the pandemic. We observed a significant increase in the rate of telemedicine visits among patients in all age groups after March 2020, especially the minor group (rate=14.36% in April 2020). In addition, the higher the age group, the lower the rate of telemedicine service. For instance, even in April 2020, the telemedicine visit rate was only 1.61% for patients aged 85 years and older.

#### Gender

**Figure 5:**
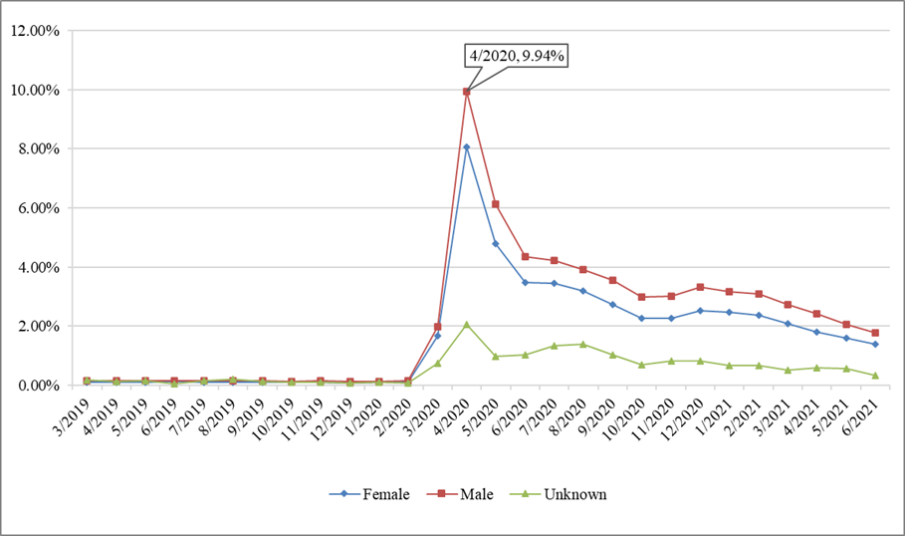
Rate of telemedicine visits by gender.

The rate of telemedicine visits increased significantly for both female and male groups, with the rate for the latter being slightly higher than for the former during the pandemic. This is likely because many female visits, such as labor and delivery, could not be conducted via telemedicine services. Moreover, males tend to have higher self-efficacy and trust in telemedicine technology compared to females and thus were more willing to switch to telemedicine services during the pandemic [14]. The trends are shown in Figure 5.

#### Race/Ethnicity

**Figure 6:**
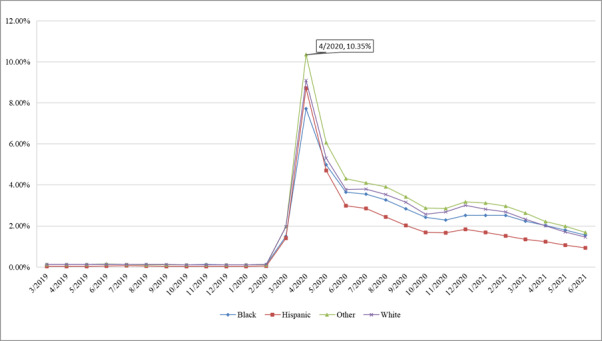
Rate of telemedicine visits by race.

The rate of telemedicine visits (peak value=10.35%) was the highest for patients in race groups other than White, Black, and Hispanic. The Alabama Medicaid data system does not contain a field for ethnicity, so Hispanics are mapped as a race. The Hispanic group had the lowest telemedicine rate throughout the study periods except in April 2020, as shown in Figure 6.

#### Rurality

**Figure 7:**
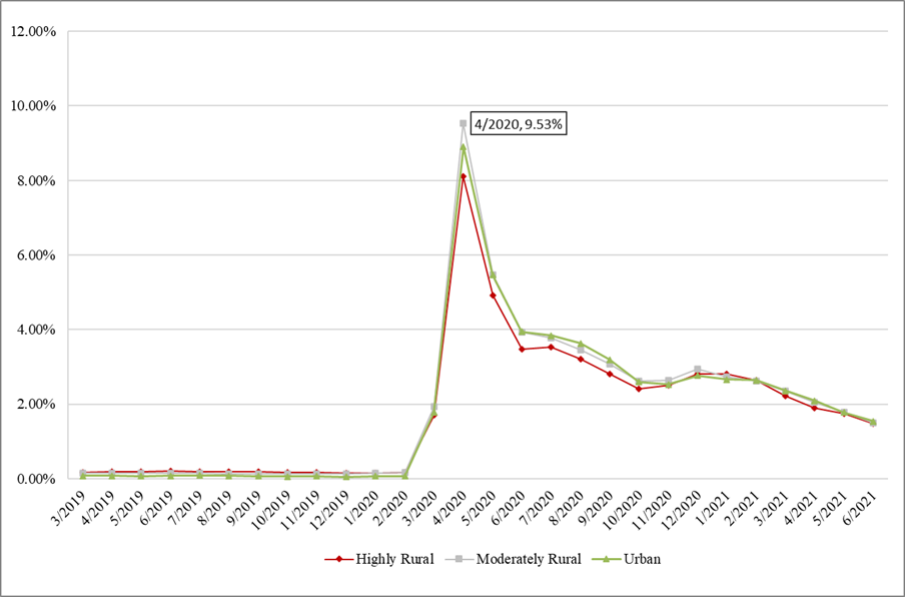
Rate of telemedicine visits by rurality.

Like any other classifications, the rates of telemedicine visits were low for all rural levels, as presented in Figure 7. The onset of the pandemic has given rise to telemedicine visits across counties with different rurality levels. The trends in rates were similar for the 3 groups. The highest rate was observed for the moderately rural group in April 2020 (rate=9.53%).

#### Procedure Type

**Figure 8:**
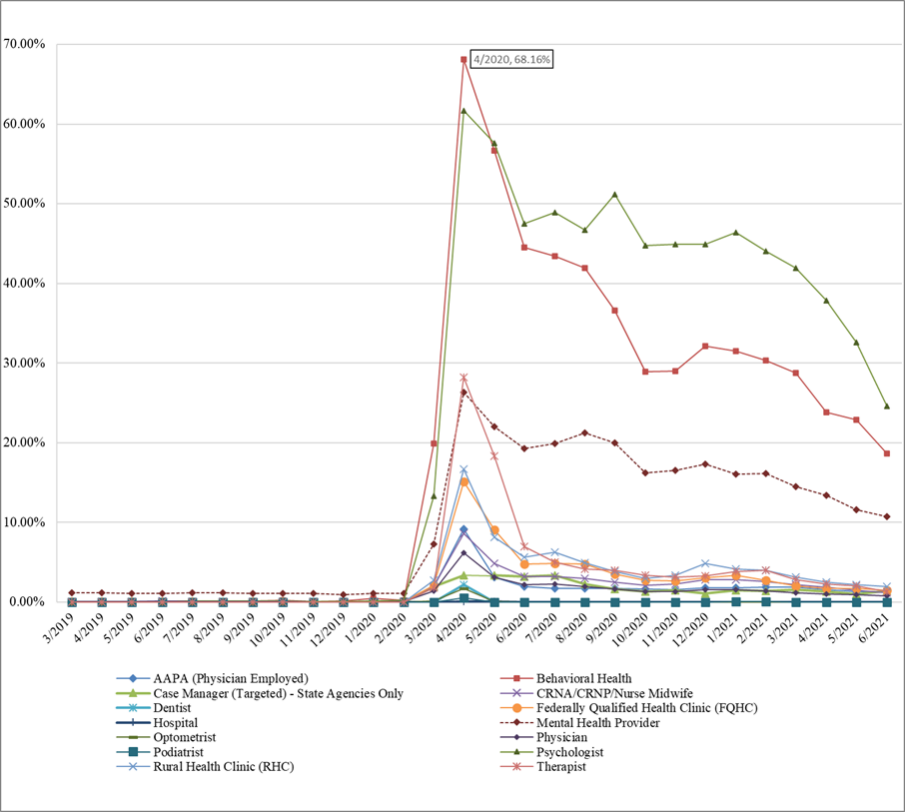
Rate of telemedicine visits by provider type.

Although the rates of telemedicine visits for behavioral health providers, psychologists, and mental health providers were not high before the onset of the pandemic, these rates have surged since the onset of the pandemic. As seen from the peak values in April 2020, 68.16% (8419/12,352) of the visits for behavioral health–related conditions, 61.67% (5850/9486) of the services provided by a psychologist, and 26.35% (55,408/210,259) of the visits for mental health–related conditions were carried out via telemedicine. These rates were maintained at a relatively high value, even after the rollout of mass vaccination. In addition, we observed a sharp increase in telemedicine rates for other services right after the onset of the pandemic; however, these rates have dropped to below 5% since August 2020. The trends are shown in Figure 8.

## Discussion

### Principal Findings

The COVID-19 pandemic introduced never-before-seen challenges to modern society. Although not directly planning for a pandemic, telehealth availability had been steadily increasing prepandemic but utilization was low. Barriers to implementing telemedicine include poor access to technical equipment, management, and reimbursement [15]. The top barriers are technology specific and could be overcome through training, change management techniques, and alternating delivery by telemedicine and personal patient-to-provider interaction. However, technology, as the major barrier, can be overcome through training, changing management techniques, and alternating delivery of services through telemedicine and personal patient-physician interactions [16]. The pandemic presented an opportunity to stress-test the underutilized telehealth modality, and the results showed that not only did the technology meet a large proportion of the needs but also a shift in society and providers toward a willingness to continue using the medium was observed. Beyond the baseline period, the COVID-19 pandemic has prompted a significant rise in the use of telemedicine for both urgent and nonurgent medical visits [17].

Although Wood et al [18] reported no significant differences in telehealth completion rates by age, sex, gender, or insurance, we found some differences in different dimensions. A telemedicine usage percentage of 57% in period 2 for 0-17-year-olds indicates a strong willingness of younger individuals to use telehealth services. The use of telemedicine by children varied by age, race, ethnicity, and recent preventive treatment, building upon previous concerns about disparities in telemedicine availability [19]. The extent to which this population directly uses telehealth versus having a parent or guardian facilitate the communications is unknown, and an area where expanded research could provide additional insights.

As a population, females experienced a higher increase in telehealth utilization compared to males. One possible reason for this could be related to the 0-17-year-old population. If mothers use telehealth for their children, then it stands to reason that they might likely do the same for themselves. Alabama Medicaid covers more than half of all births annually in Alabama, so the overall population contains a large number of females and younger persons. According to Patel et al [20], rapid telemedicine expansion can be particularly complex for pediatric patients, but approaches that satisfy privacy, security, and convenience will effectively increase pediatric enrollment capacity for telemedicine. Although telemedicine appears to be feasible and acceptable for clinical patients, questions about confidentiality, quality of care, and health disparities remain unanswered, so clinical guidelines are needed to guide best practices [21].

All races experienced a uniform increase in telehealth utilization, but the Black/African American community experienced the least in terms of percentage of increase. Research into racial disparities in telehealth by Rivera et al [22] in 2021 echoed these results by showing that African Americans are less likely to use telehealth and online services compared to Caucasians. In addition, according to Wegermann et al [23], there is still a gap in the growth of teletherapy relative to others for vulnerable populations, including those who are older, are non-Hispanic Black individuals, or have Medicare/Medicaid health insurance [23]. However, the results of this study are at odds with what Campos-Castillo and Anthony [24] found in that their data showed that African American respondents are more likely than Caucasians to report using telehealth because of the pandemic, particularly when perceiving the pandemic as a minor threat to their own health. Geographic differences could also have significant influences on racial disparities in telehealth utilization.

Although the Hispanic community experienced a large 42-fold increase, this was in part due to the fact that there was such low utilization (49 average monthly) during the prepandemic period. As for the percentage of the modality of visit type, telehealth was the lowest for Hispanics in all months examined except for April 2020, when they briefly passed the Black community by a small margin. As noted in multiple studies, telehealth access and utilization among Hispanics are inhibited by trust barriers, awareness, and eHealth literacy [25].

The willingness of younger patients to use telehealth, coupled with higher utilization rates among women, provides a positive glimpse into the future of telehealth. Racial and ethnic disparities in use due to many factors continue to exist, but the systemwide increase across all races/ethnicities also shows significant potential for sustained use. The types of services that the current set of available technologies can facilitate, however, provide insights into their usage rates.

When examining provider type, behavioral health and psychologists’ services via telemedicine experienced the highest levels during the second phase, with over 60% of both services moving to the modality. Telehealth is well equipped to provide voice and video communications, which are some of the key aspects needed for these types of services. The next highest 2 were mental health services and therapists, a continued indication that the overall set of mental health services is most easily transferred to telemedicine. Dubin et al [26] found an almost 2-fold increase in the use of telemedicine by urologists, indicating that they have the ability to adopt and adapt telemedicine into their practice, but the barriers involved in the telemedicine technology itself still prevent many from taking advantage of it [26].

Although all services have diminished since their peak in the shutdown period of the second phase of the pandemic, behavioral health, psychologists’ services, and mental health providers showed continued high utilization post-large-scale vaccine availability ranging from 10%-25%. All other services, including therapists, dropped back to below 5% telemedicine usage after vaccine availability. This is a possible indication that although telemedicine works for many services, in-person service is preferable to telemedicine. The study conducted by Smrke et al [27] reported the benefits of telemedicine for patients and clinicians in the long run since it can change cancer treatment delivery, particularly for patients with rare cancers who reside far away from expert centers.

Alhajri et al [28] found that video consultation should be frequently used in remote clinical consultation for acute conditions but that audio consultation is comparable in providing remote follow-up care for patients with chronic conditions and that audio consultation may greatly increase geographically accessible telemedicine services. Targeted efforts may be required during video visits to address patient populations that are older or have lower levels of knowledge [29]. At the same time, smartphone technology can serve as an extension of telemedicine, enabling the future of telehealth practices [30]. According to Orrange et al [31], patients’ satisfaction using telemedicine is affected by their level of confidence in physicians and visit-related factors. The aims of improving access to treatment while avoiding overuse and fraud should be balanced in telemedicine policy, both in terms of regulation and in terms of payment [32].

In terms of rurality, a uniform increase in telehealth utilization was observed across highly rural, moderately rural, and urban counties, with moderately rural counties reaching the highest overall utilization rate. Research conducted by Chu et al [10] documented an increase in the adoption of telemedicine in rural and remote areas, but the use of telemedicine increased in urban and less rural populations during the COVID-19 pandemic. The observed tight correlation of telehealth trends across all types of counties indicates a significantly uniform willingness to use telehealth and overall consistency in the availability of telehealth. Studies such as Breton et al [33] indicate that mobility issues and patients living in remote areas could negatively impact telehealth utilization, but results from this analysis did not find significant access issues, even in the most rural counties. This could be attributed to the more widespread availability of high-speed mobile data services. To preserve the long-term viability of telemedicine programs in the aftermath of the COVID-19 pandemic, persuading third parties to continue to fund these services is necessary [34]. The future of telemedicine will also require addressing access barriers for vulnerable populations, such as people with disabilities, by making significant, long-term changes in technology, regulatory and legislative infrastructure, and customized solutions that meet the unique needs of patients and health systems [35].

### Limitations

There are multiple factors that influence patient and practitioner willingness to use telemedicine, including connectivity, familiarity, and reimbursement policies, among others. The results of this study are likely applicable to states with similar demographics and rurality as Alabama, but broader applicability across the United States is questionable. However, Alabama has a high degree of rurality, poverty, and generally lower metrics in many socioeconomic factors, so if a technology such as telehealth can work in rural Alabama amongst its Medicaid population, then as long as connectivity exists elsewhere, we see no reasons, if policies allow, that similar results cannot be observed in other regions. In addition, it was reported that there were substantial differences in telemedicine completion rates among commercial insurance, Medicare, and Medicaid [36]. We only examined patients with a particular insurance type (ie, Medicaid) in this study, and researchers can also conduct similar analyses on telemedicine adoption of patients with other insurance types. Lastly, the study was descriptive in nature, which did not allow for the controlling of confounding factors. As a result, it is difficult to justify if any of the changes seen in a particular variable (ie, rurality) are not a product of other demographics (ie, if a particular demographic group of people may tend to live in a more rural area). Future studies may build multiple regression models to systematically investigate the factors that could influence telemedicine adoption.

### Conclusion

The pandemic has had a jarring and brutal impact in terms of loss of life, economic stress and despair, and many other negative aspects. If a silver lining were to exist, though, from health care access Wand availability perspectives, it would be in the form of telemedicine adoption and utilization. Within the Alabama Medicaid population, telehealth services have proven to be extremely viable and have withstood the large-scale availability of vaccines to continue to be a modality of choice for health care in both rural and urban areas. Telemedicine is likely to continue to play an integral role in health care, and as a first step toward increasing the use of telemedicine, health care systems should focus on improving patient portal usage for better access to telemedicine services [37].

The potential benefits of sustained large-scale utilization of telehealth, especially in rural areas, are quite significant, yet concerns exist. As noted by Shachar et al [38], although telehealth may increase access, safety and privacy concerns are still common among users. In a similar vein, Bokolo [39] called for stakeholders and policymakers to confront the social, organizational, and technological determinants that are barriers to the increased adoption of telehealth. Although all of these concerns are valid and still exist, the pandemic triggered an interesting test of the telehealth systems nationwide, and the response was shown to be promising. Even in a state such as Alabama, with high rurality and high poverty, telehealth has not only shown to be an effective stop-gap measure but also has continued to show increased utilization postvaccine availability. The noted disparities among races with lower utilization rates among Black and Hispanic communities, coupled with the difference in usage amongst urban versus highly rural areas, stand as opportunities for increased focus in the future.

## Appendix

Multimedia Appendix 1 Supplementary Table S1.

Multimedia Appendix 2 Supplementary Table S2.

## Abbreviations

IDA: Institute of Data and Analytics
