# Supplementary material for: Pandemic-Triggered Adoption of Telehealth in Underserved Communities: Descriptive Study of Pre- and Postshutdown Trends
Source: J Med Internet Res. 2022 Jul 15;24(7):e38602. doi: 10.2196/38602 (PMC9290332; doi:10.2196/38602)
Supplement: Multimedia Appendix 1 [file jmir_v24i7e38602_app1.docx]

**Table S1.** Monthly telemedicine and nontelemedicine Medicaid claims grouped by period and patient demographics.

| Variables | | Monthly nontelemedicine Medicaid claims, n (%) | | | Trend | Monthly telemedicine Medicaid claims, n (%) | | | Trend |
| --- | --- | --- | --- | --- | --- | --- | --- | --- | --- |
|  |  | Period 1 | Period 2 | Period 3 |  | Period 1 | Period 2 | Period 3 |  |
|  |  |  |  |  |  |  |  |  |  |
| **Age (years), *P*<.001** | | | | | | | | | |
|  | 0-17 | 1,621,283 (41.30) | 1,073,173 (40.06) | 1,500,784 (44.32) |  | 1733 (37.39) | 51,071 (53.70) | 35,601 (55.89) |  |
|  | 18-29 | 462,397 (11.78) | 332,697 (12.42) | 444,775 (13.14) |  | 457 (9.87) | 9868 (10.38) | 6693 (10.51) |  |
|  | 30-39 | 345,943 (8.81) | 249,813 (9.32) | 312,790 (9.24) |  | 557 (12.01) | 9929 (10.44) | 6617 (10.39) |  |
|  | 40-49 | 316,603 (8.06) | 219,858 (8.21) | 268,471 (7.93) |  | 578 (12.48) | 7892 (8.30) | 5153 (8.09) |  |
|  | 50-64 | 680,963 (17.35) | 460,029 (17.17) | 525,477 (15.52) |  | 1000 (21.57) | 12,617 (13.27) | 7795 (12.24) |  |
|  | 65-74 | 284,218 (7.24) | 197,950 (7.39) | 195,061 (5.76) |  | 236 (5.09) | 2501 (2.63) | 1338 (2.10) |  |
|  | 75-84 | 141,137 (3.60) | 95,784 (3.58) | 92,449 (2.73) |  | 59 (1.28) | 842 (0.88) | 376 (0.59) |  |
|  | 85+ | 73,325 (1.87) | 49,759 (1.86) | 46,122 (1.36) |  | 14 (0.31) | 388 (0.41) | 129 (0.20) |  |
| **Gender, *P*<.001** | | | | | | | | | |
|  | Female | 2,399,426 (61.12) | 1,649,587 (61.57) | 2,088,520 (61.68) |  | 2400 (51.79) | 53,068 (55.80) | 35,260 (55.35) |  |
|  | Male | 1,517,497 (38.65) | 1,020,126 (38.08) | 1,282,413 (37.87) |  | 2223 (47.98) | 41,945 (44.10) | 28,377 (44.55) |  |
|  | Unknown | 8945 (0.23) | 9351 (0.35) | 14,996 (0.44) |  | 11 (0.24) | 94 (0.10) | 65 (0.10) |  |
| **Race/ethnicity, *P*<.001** | | | | | | | | | |
|  | Black | 1,472,153 (37.50) | 1,009,310 (37.67) | 1,244,880 (36.77) |  | 1808 (39.01) | 33,234 (34.94) | 23,437 (36.79) |  |
|  | Hispanic | 114,738 (2.92) | 83,240 (3.11) | 121,757 (3.60) |  | 49 (1.06) | 2100 (2.21) | 1317 (2.07) |  |
|  | Other | 671,783 (17.11) | 466,643 (17.42) | 608,547 (17.97) |  | 796 (17.17) | 18,772 (19.74) | 12,792 (20.08) |  |
|  | White | 1,666,694 (42.45) | 1,119,461 (41.79) | 1,409,934 (41.64) |  | 1981 (42.75) | 40,991 (43.10) | 26,152 (41.05) |  |
| **Rurality, *P*<.001** | | | | | | | | | |
|  | High | 1,236,296 (31.49) | 828,028 (30.91) | 1,025,477 (30.29) |  | 2145 (46.28) | 27,939 (29.38) | 18,370 (28.84) |  |
|  | Moderate | 713,244 (18.17) | 489,683 (18.28) | 631,993 (18.67) |  | 1025 (22.11) | 17,849 (18.77) | 11,990 (18.82) |  |
|  | Low | 1,974,916 (50.31) | 1,360,294 (50.77) | 1,726,747 (51.00) |  | 1464 (31.58) | 49,288 (51.82) | 33,308 (51.29) |  |
