# Supplementary material for: Pandemic-Triggered Adoption of Telehealth in Underserved Communities: Descriptive Study of Pre- and Postshutdown Trends
Source: J Med Internet Res. 2022 Jul 15;24(7):e38602. doi: 10.2196/38602 (PMC9290332; doi:10.2196/38602)
Supplement: Multimedia Appendix 2 [file jmir_v24i7e38602_app2.docx]

**Table S2.** Monthly telemedicine and nontelemedicine Medicaid claims grouped by period and provider type.

| Provider type | Monthly nontelemedicine Medicaid claims (*P*<.001), n (%) | | | Trend | Monthly telemedicine Medicaid claims (*P*<.001), n (%) | | | Trend |
| --- | --- | --- | --- | --- | --- | --- | --- | --- |
|  | Period 1 | Period 2 | Period 3 |  | Period 1 | Period 2 | Period 3 |  |
|  |  |  |  |  |  |  |  |  |
| American Academy of Physician Associates (AAPA)–employed physicians | 23,264 (0.74) | 15,387 (0.73) | 21,853 (0.79) |  | 0 | 355 (0.41) | 300 (0.50) |  |
| Behavioral health | 11,081 (0.35) | 8000 (0.38) | 14,764 (0.54) |  | 18 (0.43) | 4883 (5.59) | 4299 (7.15) |  |
| Case manager (targeted) | 33,603 (1.07) | 22,280 (1.06) | 23,112 (0.84) |  | 10 (0.24) | 514 (0.59) | 327 (0.54) |  |
| Certified registered nurse anesthetist (CRNA)/certified registered nurse practitioner (CRNP)/nurse/midwife | 271,499 (8.67) | 189,631 (9.04) | 262,797 (9.53) |  | 206 (4.95) | 6072 (6.95) | 4374 (7.28) |  |
| Dentist | 230,189 (7.35) | 182,720 (8.71) | 288,609 (10.47) |  | 0 | 25 (0.03) | 4 (0.01) |  |
| Federally qualified health clinic (FQHC) | 141,790 (4.53) | 93,818 (4.47) | 126,486 (4.59) |  | 83 (2.00) | 4404 (5.04) | 2224 (3.7) |  |
| Hospital | 170,595 (5.45) | 105,565 (5.03) | 103,996 (3.77) |  | 1 (0.01) | 18 (0.02) | 4 (0.01) |  |
| Mental health | 262,456 (8.39) | 155,685 (7.42) | 185,949 (6.75) |  | 3010 (72.33) | 34,648 (39.64) | 26,415 (43.96) |  |
| Optometrist | 75,384 (2.41) | 50,065 (2.39) | 70,023 (2.54) |  | 0 | 22 (0.02) | 2 (0.00) |  |
| Physician | 1,681,678 (53.73) | 1,117,353 (53.29) | 1,438,575 (52.18) |  | 821 (19.73) | 23,245 (26.6) | 13,408 (22.31) |  |
| Podiatrist | 6788 (0.22) | 3152 (0.15) | 3100 (0.11) |  | 0 | 1 (0.001) | 1 (0.001) |  |
| Psychologist | 17,821 (0.57) | 5551 (0.26) | 8173 (0.30) |  | 10 (0.23) | 4659 (5.33) | 3996 (6.65) |  |
| Rural health clinic | 132,971 (4.25) | 91,530 (4.37) | 126,822 (4.60) |  | 2 (0.04) | 5047 (5.77) | 3099 (5.16) |  |
| Therapist | 70,612 (2.26) | 56,159 (2.68) | 82,554 (2.99) |  | 1 (0.02) | 3503 (4.01) | 1645 (2.74) |  |
